# Expression patterns of *FLAGELLIN SENSING 2* map to bacterial entry sites in plant shoots and roots

**DOI:** 10.1093/jxb/eru366

**Published:** 2014-09-09

**Authors:** Martina Beck, Ines Wyrsch, James Strutt, Rinukshi Wimalasekera, Alex Webb, Thomas Boller, Silke Robatzek

**Affiliations:** ^1^The Sainsbury Laboratory, Norwich Research Park, Norwich NR4 7UH, UK; ^2^Zürich-Basel Plant Science Center, University of Basel, Department of Environmental Sciences, Botany, Basel, Switzerland; ^3^Department of Plant Sciences, University of Cambridge, Downing Street, Cambridge CB2 3EA, UK

**Keywords:** Bacteria, flagellin, flg22, pattern recognition receptor, promoter expression, stomata.

## Abstract

Pathogens can colonize all plant organs and tissues. To prevent this, each cell must be capable of autonomously triggering defence. Therefore, it is generally assumed that primary sensors of the immune system are constitutively present. One major primary sensor against bacterial infection is the FLAGELLIN SENSING 2 (FLS2) pattern recognition receptor (PRR). To gain insights into its expression pattern, the *FLS2* promoter activity in β-glucuronidase (GUS) reporter lines was monitored. The data show that *pFLS2*::GUS activity is highest in cells and tissues vulnerable to bacterial entry and colonization, such as stomata, hydathodes, and lateral roots. GUS activity is also high in the vasculature and, by monitoring Ca^2+^ responses in the vasculature, it was found that this tissue contributes to flg22-induced Ca^2+^ burst. The *FLS2* promoter is also regulated in a tissue- and cell type-specific manner and is responsive to hormones, damage, and biotic stresses. This results in stimulus-dependent expansion of the *FLS2* expression domain. In summary, a tissue- and cell type-specific map of *FLS2* expression has been created correlating with prominent entry sites and target tissues of plant bacterial pathogens.

## Summary

Expression of the flagellin receptor *FLS2* is regulated in a cell/tissue-specific and stress-induced manner that correlated with sites of bacterial infection. The vasculature expresses *FLS2* and responds to flagellin.

## Introduction

Plant pathogens use a variety of different strategies to invade their hosts, which are tightly associated with the lifestyle of the pathogen as well as with plant development (Faulkner and Robatzek, 2012). The general aim of a pathogen is to invade and access plant tissues where it can find nutrients for its own development. Bacterial phytopathogens typically try to reach the apoplastic space between cells where they can multiply and reprogramme host metabolism by the injection of bacterial effectors into the extra- and intracellular space. During a susceptible interaction, as observed between *Arabidopsis thaliana* and *Pseudomonas syringae*, the Gram-negative bacterium enters the host tissue (typically leaves) via natural openings (stomata) or wound sites, from where it propagates in the apoplastic spaces, causing water-soaked, chlorotic (and later also necrotic) lesions (Preston, 2000).

Lacking a circulatory system and specialized immune cells, plants depend upon the ability of every cell to recognize potentially pathogenic microbes and initiate immunity. For this, plants exploit cell surface-localized pattern recognition receptors (PRRs), which allow the detection of conserved microbial molecules, so-called pathogen-associated molecular patterns (PAMPs) (Boller and Felix, 2009). In the case of immunity against bacterial pathogens, a major PRR is the receptor kinase FLAGELLIN SENSING 2 (FLS2) which recognizes bacterial flagellin through its conserved elicitor-active epitope flg22 (Gomez-Gomez *et al.*, 1999). Studies show that flg22 triggers defence responses in whole seedlings, leaves, and roots (Zipfel *et al.*, 2004; Millet *et al.*, 2010; Jacobs *et al.*, 2011). This suggests that the receptor is expressed in these tissues, which is consistent with findings of mRNA expression studies and FLS2–green fluorescent protein (GFP) imaging (Gomez-Gomez and Boller, 2000; Robatzek *et al.*, 2006). These observations generally imply that defence components such as FLS2 might be constitutively expressed, but this might lead to an unwanted activation of defence responses which can negatively impact plant processes such as growth. A typical response, which can be observed for plants that are exposed long term to flagellin, is the reduction in plant growth, due to a defined trade-off between immune and hormonal signalling (Gomez-Gomez *et al.*, 1999; Navarro *et al*., 2006, 2008; Lozano-Duran *et al.*, 2013).

Publicly available gene expression data (Arabidopsis eFP browser; Faulkner and Robatzek, 2012) revealed that *FLS2* is not expressed at similar levels throughout the plant. For example, *FLS2* does not have measurable expression in root cells, despite flg22 triggering some defence responses in this organ (Millet *et al.*, 2010; Jacobs *et al.*, 2011). In leaves, FLS2 exhibits a more specific cellular function since flg22 perception seems to play a predominant role in stomatal immunity (Zipfel *et al.*, 2004; Zeng and He, 2010). Recent studies showed that *FLS2* transcriptional activation depends on ethylene signalling involving binding of the transcription factors ETHYLENE-INSENSITIVE 3 (EIN3) and ETHYLENE-INSENSITIVE3-LIKE 1 EIL1 (Boutrot *et al.*, 2010; Mersmann *et al.*, 2010), and is positively regulated by its own ligand and other PAMPs (Zipfel *et al.*, 2004, 2006). These observations indicate that *FLS2* expression is under spatio-temporal control, but the extent to which the transcription of *FLS2* is regulated remains unknown.

Here, it is demonstrated that the *FLS2* promoter is active in a cell type- and tissue-specific manner and is up-regulated in response to hormones and stress. Using transgenic *Arabidopsis* plants producing β-glucuronidase (GUS) under the control of the *FLS2* promoter, *pFLS2*::GUS activity was detected in all organs, with the highest levels found in hydathodes, stomata, and the vasculature, representing prominent entry sites and target tissues of bacteria in plants. Tissue-specific Ca^2+^ measurement shows that the vasculature is responsive to flg22. Detailed imaging revealed, furthermore, that FLS2 is present in roots but restricted to outgrowing lateral roots (LRs) and the inner central cylinder, suggesting a specific role for FLS2 in these tissues. Hormones, wounding, and abiotic and biotic stress can differentially activate *pFLS2*::GUS in specific tissue layers. Altogether, this study provides a detailed expression map of a major plant immune receptor and reveals spatio-temporal control of the *PRR* promoter activity for optimal plant defences under pathogen attack.

## Materials and methods

### Plant materials and growth conditions

The *A. thaliana* transgenic plants used in this study (accession Columbia-0, if not otherwise indicated) were *fls2* (Zipfel *et al.*, 2004) and *DR5*::GFP (Benkova *et al.*, 2003); courtesy of J. Friml. For microscopy, stress treatments, and developmental studies, seedlings were grown for 6–8 d on sterile 1× Murashige and Skoog (MS) plates supplemented with 1% sucrose and 0.8% phytoagar (w/v) under 16h light at 22 °C. For Ca^2+^ measurement, Col-0 35S:AEQ and the GAL4-mediated vascular enhancer trap line KC274 were used (Marti *et al.*, 2013). Seeds were surfaced sterilized and sown on 0.5× MS medium with 0.8% agar (w/v). Seedlings were grown in long days at 19 °C at light intensity 50 μmol m^–2^ s^–1^ (Sanyo MLR30 growth cabinet) for 12 d. For non-sterile conditions used in developmental studies, plants were grown for 2–8 weeks on soil under controlled environments (12h light, 22 °C, and 60% humidity).

### Gene constructs and plant transformation

The promoter of *FLS2* (988bp) was used from *pFLS2*::FLS2–GFP (Robatzek *et al.*, 2006) and fused to the *GUS* gene, which was isolated from pGUS Topo via *Bam*HI and *Hin*dIII restriction sites and inserted into *pFLS2*::pCAMBIA2300, resulting in *pFLS2*::GUS-pCAMBIA2300. Col-0 plants were transformed via *Agrobacterium*-mediated transformation with the floral dipping method (Clough and Bent, 1998). Transformants were selected for kanamycin resistance. The experiments were repeated in two independent transgenic lines of the T_3_ generation.

### GUS staining

All samples were processed according to the method described by De Block and Debrouwer (1992), with 1mM 5-bromo-4-chloro-3- indolyl-d-glucuronide (X-Gluc) in staining buffer [0.1M NaH_2_PO_4_, 0.1M Na_2_HPO_4_, 10mM EDTA, 2mM FeK_3_(CN)_6_, 2mM FeK_4_(CN)_6_·3 H_2_O, pH 7.0, 0.1% (v/v) Triton X-100] at room temperature for 2–18h. Samples were fixed and destained with ethanol/acetic acid (50% v/v). Specimens were examined and documented using a Leica M165 FC stereomicroscope.

### Embedding and sectioning

Tissue was fixed in 2.5% glutaraldehyde or 4% paraformaldehyde, followed by a ethanol series of 30, 50, 70, 90, and 100%, for 30min each. Pre-infiltration of the tissue was done for 2h with 50:50 (v/v) ethanol:Technovit^®^7100 (Heraeus-Kulzer, Germany) base liquid. The preparation solution (Technovit^®^7100; see the supplier’s embedding protocol) was infiltrated and tissue samples were left for polymerization. Samples were sectioned to 10 μm thickness by using an Ultracut E ultramicrotome (Reichert-Jung, Germany).

### Microscopy

Standard confocal laser microscopy was performed using a Leica SP5laser point scanning microscope. GFP/propidium iodide was excited using the 488nm argon laser, and fluorescence emissions were captured between 500nm and 550nm for GFP and between 580nm and 640nm for propidium iodide. Seedlings were incubated for 20min in 10 μg ml^–1^ propidium iodide solution.

### Stress treatments

The chemicals were diluted in half-strength MS medium to their respective working solutions: 10 μM flg22 (10mM in dH_2_O), 50 μM salicylic acid [SA; 100mM in dimethylsulphoxide (DMSO)], 1mM H_2_O_2_ (1.5M), 10 μM 1-aminocyclopropane-1-carboxylic acid (ACC; 10mM in dH_2_O), and 10 μM indole acetic acid (IAA; 100mM in dH_2_O). Half-strength MS medium was used as mock treatment. For each treatment, seedlings (8–10 d after germination) were transferred from agar plates and incubated in the respective solutions for 48h under 16h light at 22 °C, followed by GUS staining. For bacterial stress and wound treatments, detached leaves of 3-to 4-week-old soil-grown plants were used. Detached leaves were submerged in 10mM MgCl_2_ (mock) or with *Pseudomonas syringae* pv. *tomato* DC3000 (*Pto* DC3000; OD 0.1) in 10mM MgCl_2_ solution, with slight shaking for 24h at room temperature. Wound stress was inflicted by a sharp needle on 10 detached leaves mounted on half-strength MS agar and left on plates for 4–6h at room temperature before staining. All stress treatments were performed on at least 10 seedlings or 10 leaves of the two independent T_3_ transgenic lines at the same developmental stage. Images show representative results of three biological repetitions.

### Ca^2+^ measurements

Seedlings grown for 12 d were supplied with half-strength MS liquid medium supplemented with 20 μM coelenterazine (Nanolight) and incubated overnight in the dark at room temperature. Luminescence measurements were performed using a FLUOstar OPTIMA plate reader (BMG LABTECH). Luminescence from single wells was measured over 35 s, and flg22 (EZBiolab) dissolved in half-strength MS was injected to a final concentration of 100nM and measured at 15 s intervals for 1200 s. Mock treatment (water, 35 s) was performed under the same conditions. At the end of the experiment, the remaining aequorin (AEQ) pool was discharged by treatment with a final concentration of 1M CaCl_2_ in 10% (v/v) ethanol. Luminescence values were converted to estimates of intracellular Ca^2+^ ([Ca^2+^]_i_) according to Fricker *et al.* (1999).

### LR growth analysis

Col-0 and *fls2* were germinated on 1× MSN plates and transferred 3 d after germination in liquid 1× MS medium with or without 1 μM flg22. After 6 d, the root length and number of LRs were determined.

### Immunoblot and ConA precipitation

A 100mg aliquot of root tissue of seedlings (Col-0) grown for 2 weeks vertically on 1× MS plates was homogenized in 0.2ml of cold IP buffer [50mM TRIS-HCl pH 8, 150mM NaCl, 1% (v/v) Nonidet P40, and protease inhibitor cocktail] and incubated for 1h at 4 °C followed by a centrifugation step (10 000 *g* for 10min, three times). The supernatant was incubated for 1h at 4 °C with concanavalin A (ConA)–Sepharose beads (Amersham Biosciences) to enrich samples for glycosylated proteins. This was used because FLS2 is highly glycosylated (Häweker *et al.*, 2010) and weakly detectable in root total extracts. The beads were collected and washed three times with ice-cold IP buffer. After denaturation in SDS–PAGE sample loading [0.35M TRIS-HCl pH 6.8, 30% (v/v) glycerol, 10% (v/v) SDS, 0.6M dithiothreitol, and 0.012% (w/v) bromophenol blue], proteins retained on the beads were eluted by SDS–PAGE sample loading buffer and separated by 7% SDS–PAGE. FLS2 was detected by immunoblot analyses with anti-FLS2 antibodies (Chinchilla *et al.*, 2006).

### MAPK activation in roots

Isolated roots of 2-week-old plants (*n*=12) were placed in dH_2_O for 16h. Flg22 at 1 μM was added for 10min and tissue (50mg per sample) was shock frozen. To the ground material, 50 μl of SDS–PAGE sample loading [0.35M TRIS-HCl pH 6.8, 30% (v/v) glycerol, 10% (v/v) SDS, 0.6M dithiothreitol, and 0.012% (w/v) bromphenol blue] was added. Total proteins were separated by electrophoresis in a 12% SDS–polyacrylamide gel and electrophoretically transferred to a polyvinylidene fluoride membrane according to the manufacturer’s instructions (Bio-Rad). Transferred proteins were detected with Ponceau-S. Polyclonal primary antibodies against phospho-p44/42 mitogen-activated protein kinase (MAPK; Cell Signaling Technologies) were used, with alkaline phosphatase-conjugated anti-rabbit as secondary antibodies. Signal detection was performed using CDPstar (Roche).

### Microarray

Landsberg *erecta* (ecotype L*er*) seedlings and *fls2-17* (Zipfel *et al.*, 2004) were grown in liquid culture under constant shaking in 1× MS medium for 21 d. Plants were mock or flg22 (10 μM, 30min) treated, and roots were harvested and stored at –80 ºC for sample preparation. Experimental conditions for RNA extraction, microarray hybridizations, and statistical analyses were performed as in Zipfel *et al.* (2004).

## Results

### *FLS2* is highly expressed in stomata, hydathodes, and wound sites in leaves

To investigate the promoter activity of *FLS2* at the tissue level, transgenic *A. thaliana* lines containing the putative promoter sequences of the *FLS2* gene fused to *GUS* were generated. An ~900bp genomic sequence upstream of the start codon of *FLS2* was used (Supplementary Fig. S1 available at *JXB* online), which was sufficient to complement fully an *fls2* mutant expressing the FLS2–GFP fusion protein (Zipfel *et al.*, 2004). *In silico* motif analysis of the promoter sequence 900bp upstream of At5g46330 revealed the presence of a TATA box motif and several *cis-*elements such as W-boxes, known binding sites of WRKY transcription factors (Supplementary Fig. S1). Two binding sites in the region were previously shown to be occupied by EIN3 and EIL1, transcription factors of the ethylene pathway mediating *FLS2* expression (Boutrot *et al.*, 2010).

By monitoring GUS accumulation in the *pFLS2*::GUS lines during plant development, it could be confirmed that the *FLS2* promoter exhibited expression in all organs examined (Supplementary Fig. S2 at *JXB* online). In 2-day-old seedlings, a clear blue staining could be detected in the developing cotyledons and root. In older seedlings, a prominent staining occurred additionally in the vascular tissue of cotyledons and the hypocotyl (Supplementary Fig. S2). At later stages of plant development, stipules, small leaf-like appendage at the bases of leaves, as well as floral and reproductive organs including petals, stamen, and the dehiscence zone in mature, conferred a clearly visible *pFLS2*::GUS expression (Supplementary Fig. S2).

**Fig. 1.**
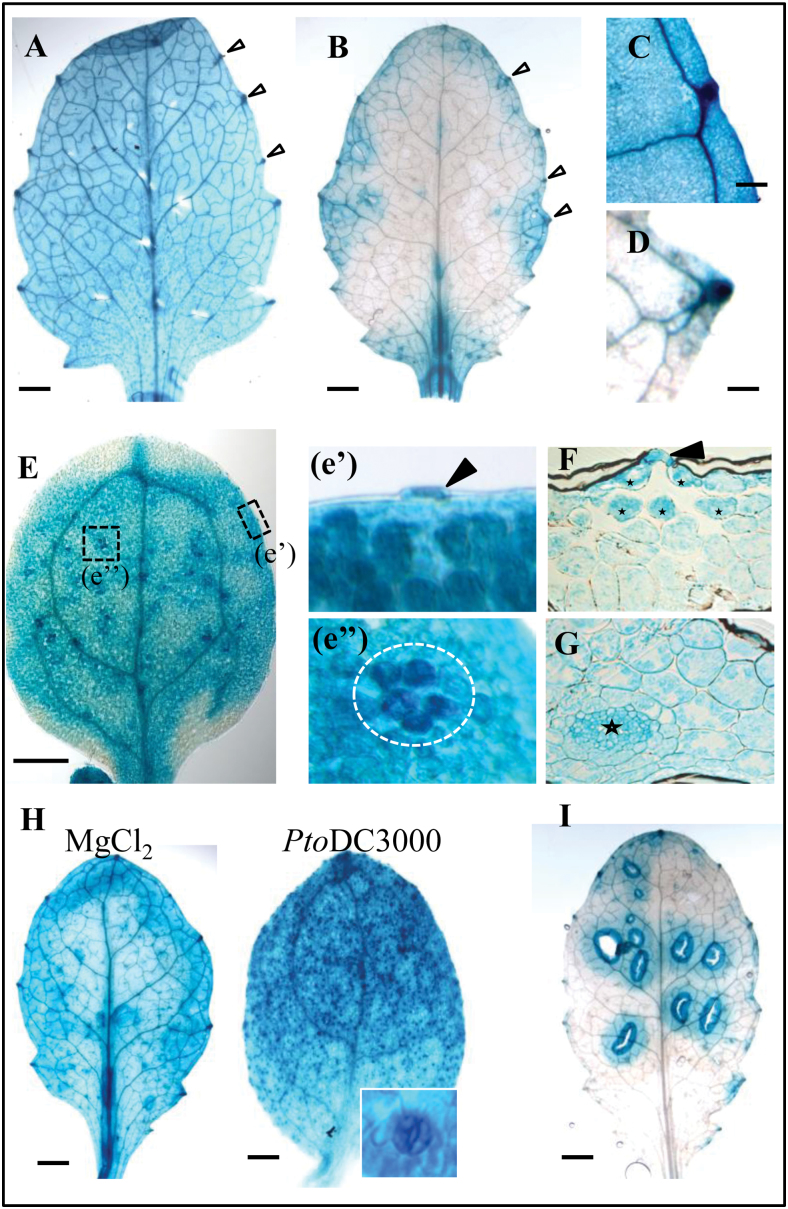
*FLS2* is differentially activated in leaves. Representative images of *pFLS2*::GUS expression. (A) First pair of true leaves. (B) Second pair of true leaves. Arrows show strong expression in hydathodes from (C) cotyledons and (D) the second pair of true leaves. (E) Promoter activity in cotyledons; dashed boxes show expression (e’) in stomata (arrow) and (e’’) a group of mesophyll cells (circle). (F) Cross-section of cotyledons shows guard cell expression (arrow) and high GUS staining in mesophyll cells surrounding the stomatal cavity (asterisks); (G) shows high expression in leaf veins (asterisk) and mesophyll. (H) *Pto* DC3000 increases promoter activity in stomata from the first pair of true leaves compared with mock (MgCl_2_) treatment. The inset shows an enlarged stoma. (I) Wound-induced GUS staining in the second pair of true leaves. (A, B, E, H, I) bar=1mm, (C, D) bar=0.1mm.

As FLS2-mediated immunity is predominantly studied in the *Arabidopsis* interaction with the leaf-infecting pathogen *Pto* DC3000, the basal *pFLS2*::GUS expression in different leaf developmental stages was studied (Supplementary Fig. S3A at *JXB* online). In cotyledons and the first pair of true leaves, the promoter expression showed a homogenous pattern throughout the leaf tissue, with higher expression levels in the vascular tissue and hydathodes (Fig. 1A, C, E). In younger leaves, GUS staining exhibited a more patchy distribution throughout the leaves (Fig. 1B; Supplementay Fig. S3B), but continuously showed a strong staining in hydathodes (Fig. 1D). At the cellular level, *pFLS2*::GUS expression was significantly visible in the mesophyll and phloem, as well as in epidermal cells, such as in the guard cells of the stomata (Fig. 1E, G). Notably, the mesophyll cells underneath the stomatal openings, forming the substomatal cavity, had clear promoter activity as revealed by cross-sectioning of leaf tissues (Fig. 1F).

The substomatal expression pattern is correlated to cells exposed to early invasion of bacteria, which enter the apoplastic space underneath stomata. To visualize the entry of bacteria in *Arabidopsis* leaves, Col-0 plants were incubated with a GFP-transformed *Pto* DC3000 strain (Supplementary Fig. S4A at *JXB* online). The GFP-labelled bacteria were clearly visible in epidermal cells and within the openings of stomata (Supplementary Fig. S4A). Bacterial accumulation was often detectable in the intercellular space of mesophyll cells directly underneath stomata (Supplementary Fig. S4A). Next it was tested whether the presence of bacteria on the leaf surface would have an influence on the *FLS2* promoter activity. Overnight incubation of 14- to 18-day-old plants with *Pto* DC3000 led to a strong visible GUS staining in stomatal guard cells in leaves and the hypocotyl (Fig. 1H; Supplementary Fig. S3D).

Bacteria also take advantage of wound sites and cracks in the epidermis to enter plant tissues, and therefore the influence of wounding on the *FLS2* promoter activity was investigated. In general, in young leaves the *pFLS2*::GUS activity was very low in the absence stimuli (Fig. 1B). By contrast, wounding of leaves led to up-regulation of the promoter around the wound sites (Fig. 1I; Supplementary Fig. S3C), which was not obvious in cotyledons and first true leaves (Supplementary Fig. S3C). All these findings reveal that high levels of *FLS2* expression in leaves occur in cells and tissues that represent natural entry sites of bacteria, or can become entry sites due to wounding.

### *FLS2* shows specific expression patterns and flg22 responses in roots

**Fig. 2.**
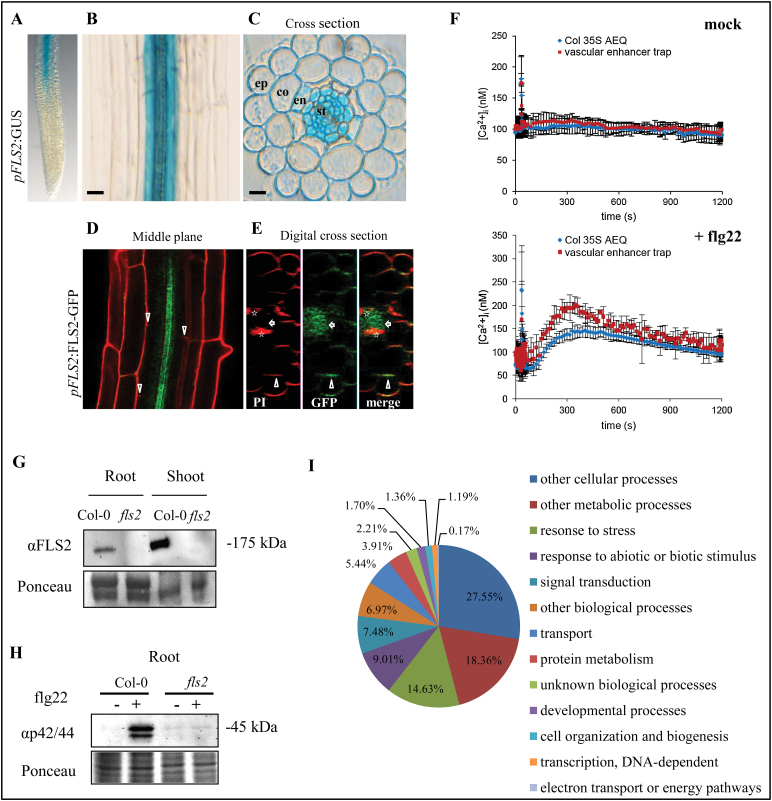
Roots exhibit specific *FLS2* expression patterns and tissue-specific responsiveness to flg22. In sterile-grown roots (8 d after germination) of *pFLS2*::GUS, the promoter activity is not present in root tips (A), but shows a high expression in the root stele (B) as revealed by root cross-section (C); bar=10 μm. (D) Confocal micrographs of *pFLS2*::FLS2–GFP show accumulation of GFP signal in the inner part of the stele (arrowheads point to inhibited uptake of propidium iodide at the endodermis; bar=10 μm. (E) Digital cross-section with plasma membrane localization of FLS2–GFP at cortical cells (arrowheads) and in the root cylinder (arrow). Autofluorescence of xylem is marked with asterisks. (F) Changes in [Ca^2+^]_i_ values in mock-treated control (water, 35 s) or in response to flg22 (100nm, 35 s) in 35S::AEQ seedlings and the vasculature enhancer trap line KC274. Luminescence was measured over 1200 s. Data are presented as means ±SD, *n*=4 (mock), *n*=6 (flg22). (G) Immunoblot of detected FLS2 protein in roots and shoots. Samples were enriched for glycosylated proteins using ConA. (H) Immunoblot detection of phosphorylated MAPK present in Col-0 after 1 μM flg22 (10min) treatment but not in *fls2*. (I) Gene ontology of enriched genes specifically up-regulated in L*er* roots after flg22 treatment (10 μM, 30min).

In roots, the *pFLS2*::GUS lines showed a basal expression in the root vascular cylinder starting at the root differentiation zone; no *GUS* expression could be observed in the root meristematic zone (Fig. 2A, B). Under sterile conditions, the highest expression was restricted to the inner cellular layers of the root, the vascular cylinder (Fig. 2B). In root cross-sections, a pronounced accumulation of GUS precipitate was observed in cells inside the endodermis (Fig. 2C) and this expression maximum correlated with a high accumulation of the *pFLS2*::FLS2–GFP fusion protein in the stele as revealed by co-staining the roots with the apoplastic tracer propidium iodide, uptake of which is blocked at the endodermis (Alassimone *et al.*, 2010) (Fig. 2D). These observations are consistent with the accumulation of the native FLS2 protein in roots as revealed by immunoblot analysis (Fig. 2G). This basal expression pattern of FLS2 in roots may protect the plant from bacterial infections of the vasculature and ultimately colonization throughout all tissues.

One of the earliest responses to PAMPs is a transient and rapid (within seconds) increase of free [Ca^2+^]_i_, which subsequently (within minutes) declines to steady-state [Ca^2+^]_i_ levels (Blume *et al.*, 2000; Ranf *et al.*, 2008). This [Ca^2+^]_i_ increase was shown to be crucial for many downstream responses. To test whether the vasculature tissue is sensitive to flg22 stimulation, the GAL4-mediated vascular enhancer trap line KC274 expressing AEQ specifically in the vasculature (Marti *et al.*, 2013) was exploited. Treatment with flg22 induced a rapid increase in [Ca^2+^]_i_ in both the vasculature-specific KC274;UAS AEQ line and in the line in which aequorin was expressed constitutively under the control of the *Cauliflower mosaic virus* (CaMV) 35S promoter (Fig. 2F). The magnitude of the reported flg22-induced increase in [Ca^2+^]_i_ was greater when AEQ was targeted specifically to the vasculature tissue in KC274 (Fig. 2F), suggesting that FLS2 in the vasculature mediates a typical early flg22 response and indicates that this tissue contributes to the source of the PAMP-induced [Ca^2+^]_i_ burst in plants.

To gain further insights into the functional relevance of the presence of FLS2 in roots, the phosphorylation of MAPKs upon flg22 elicitation was studied. Immunoblot analysis revealed a specific flg22-induced activation of MAPK in root tissue of Col-0 but not *fls2*, demonstrating that FLS2 in roots activates similar signalling responses to those shown for leaf tissues (Fig. 2H).

The root’s response to flg22 elicitation was further explored on a more global scale, and whole-transcriptome expression analysis was performed. Sterile grown seedlings (L*er*) were mock and flg22 treated, and roots were harvested after 30min. ATH1 microarray expression analysis revealed flg22-regulated genes overlapping with those identified from whole-seedling expression analysis (Zipfel *et al.*, 2004), but also identified ~75 genes specifically up-regulated in roots (Fig. 2I). Fifty-three of these genes showed a >2.5-fold induction after flg22 treatment (Supplementary Table S1 at *JXB* online). Sixty-five of these genes have their highest expression values during root development (eFP Browser http://bar.utoronto.ca/efp/cgi-bin/efpWeb.cgi), which confirmed the enrichment for root-specific processes (Supplementary Table S1). After flg22 elicitation, in the roots transcriptional changes were seen of genes with roles in hormone and stress signalling, such as auxin- and ethylene-mediated pathways (AT1G59500, AT5G65600, AT1G72360, and AT5G46080), root and LR development (AT4G31500 and AT5G13080), or signalling and defence pathways (AT2G17060 and AT3G21650) (Table 1). Taken together, the data show that not only *FLS2* promoter activity, but also the functional protein, is present in roots. Root-specific activation of FLS2 reveals a subset of genes which are specifically enriched after flg22 treatment, indicating additional functions of this receptor in roots.

**Table 1.** flg22-induced genes in roots: candidates with maximum expression in roots

	Gene	flg22 fold induction	Maximum expression level	Annotation	Biological process
Hormone and stress signalling	AT1G59500	6.67	3915.41^*a*^	GH3.4; indole-3-acetic acid amido synthetase	Auxin homeostasis, response to auxin stimulus
	AT5G65600	5.3	1132.13^*b*^	Legume lectin family protein/ protein kinase family protein	Protein phosphorylation, response to ethylene stimulus
	AT1G08050	4.46	2268.52^*b*^	Zinc finger (C3HC4-type RING finger) family protein	MAPK cascade, abscisic acid-mediated signalling pathway, cell communication
	AT5G11920	4.2	8344.6^*b*^	AtcwINV6 (6-&1-fructan exohydrolase)	Carbohydrate metabolic process, regulation of hydrogen peroxide metabolic process
	AT1G15670	4.05	14417.2^*b*^	Kelch repeat-containing F-box family protein	Negative regulation of cytokinin-mediated signalling pathway
	AT5G67340	3.94	3905.65^*b*^	Armadillo/beta-catenin repeat family protein	Endoplasmic reticulum–nucleus signalling pathway, MAPK cascade, negative regulation of defence response
	AT1G72360	3.76	11466.3^*b*^	Ethylene-responsive element- binding protein	Cellular response to ethylene stimulus, regulation of transcription
	AT3G28580	3.76	8339.31^*b*^	AAA-type ATPase family protein	Response to abscisic acid stimulus, response to ethylene stimulus
	AT5G46080	3.62	890.14^*b*^	Protein kinase family protein	Ethylene biosynthetic process, protein phosphorylation
	AT5G01550	3.08	1208.89^*b*^	LECRKA4.2 (LECTIN RECEPTOR KINASE A4.1)	Abscisic acid-mediated signalling pathway, protein phosphorylation, response to chitin
	AT3G13100	2.66	2294.74^*b*^	ATMRP7; ATPase	Response to other organisms, salicylic acid biosynthetic process
Root development					
	AT4G31500	3.44	17621.3^*b*^	CYP83B1 (CYTOCHROME P450 MONOOXYGENASE)	Adventitious root development, callose deposition in cell wall during defence response
	AT1G67980	3.42	1164.11^*b*^	CCoAMT; caffeoyl-CoA O-methyltransferase	Lignin biosynthetic process
	AT3G45960	2.83	1535.55^*b*^	ATEXLA3 (*Arabidopsis thaliana* expansin-like a3)	Plant-type cell wall loosening, plant-type cell wall organization
	AT5G13080	2.58	3789.3^*b*^	WRKY75; transcription factor	Cellular response to phosphate starvation, lateral root development, response to ethylene stimulus
Signalling/defence					
	AT2G17060	3.79	561.85^*b*^	Disease resistance protein (TIR- NBS-LRR class)	Defence response, signal transduction
	AT4G28350	3.66	1223.16^*b*^	Lectin protein kinase family protein	Defence response to fungus, protein phosphorylation, response to chitin
	AT1G64400	3.09	2202.11^*b*^	Long-chain-fatty-acid–CoA ligase	Defence response to insect, fatty acid biosynthetic process
	AT3G21650	2.74	900.6^*b*^	Serine/threonine protein phosphatase 2A (PP2A)	Signal transduction

^*a*^ Lateral root.

^*b*^ Root.

### *FLS2* is highly expressed in emerging lateral roots

In soil, roots are exposed to a variety of microorganisms, both pathogenic and beneficial. Interestingly, when plants were grown under non-sterile conditions, an up-regulation of *FLS2* promoter expression was observed in the endodermis and cortical cells but not in epidermal cells, showing that the *pFLS2*::GUS expression in roots is not restricted to the vascular cylinder but can expand at least to the cortical cell layer (Supplementary Fig. S5A, B at *JXB* online).

**Fig. 3.**
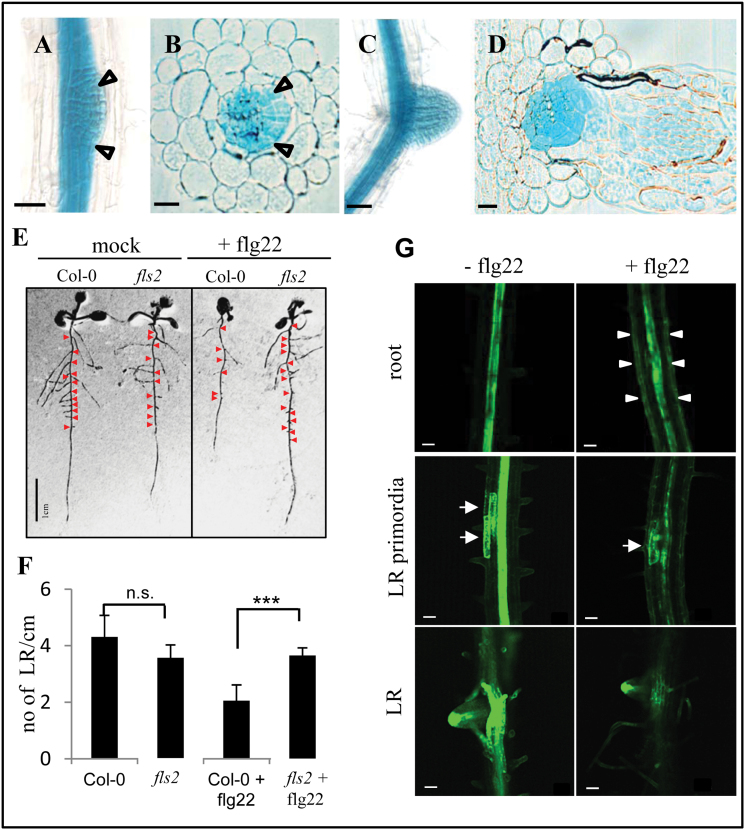
Flg22 affects growth of *FLS2*-expressing lateral roots and auxin distribution. (A) *pFLS2*::GUS seedlings (10 d after germinationg) show prominent GUS staining in outgrowing lateral roots (LRs) (arrows); bar=50 μm. (B) Cross-section of LR outgrowth (arrows); bar=10 μm. (C) Promoter activity is present in a developed LR; bar=50 μm. (D) Cross-section of a developed LR; bar=10 μm. (E) Col-0 and *fls2* seedlings 12 d after germination with and without flg22 (1 μM) treatment; red arrows indicate LRs. (F) Graph showing quantification of LR per cm root length in Col-0 and *fls2* seedlings with and without flg22 treatment (1 μM); bars represent the average of three independent experiments; error bars represents the SD; statistical significance is represented by Student’s *t*-test (*P*-value >0.001). (G) Confocal micrographs show roots of *DR5*:GFP transgenic seedling roots (10 d after germination) incubated for 72h with or without flg22 (1 μM); arrowheads indicate GFP signals in epidermal cells of flg22-treated seedlings; middle and bottom panels depict different developmental stages of LR formation along the axis of 10-day-old roots; arrows indicate *DR5*:GFP signals marking LR primordia; bar=50 μm.

This expansion of the promoter activity to different tissues also became apparent during the developmental process of LR growth. The *pFLS2*::GUS lines exhibited significant staining in the LR primordia and outgrowing LRs (Fig. 3A–D). When they reached a certain developmental stage, the promoter activity was restricted again to the vascular cylinder of the developed LR and no staining was found in the tip of the LR, similarly to what was observed for the primary root tip (data not shown). Outgrowing LRs provide prominent entry points of bacterial pathogens as the outgrowth from the pericycle to the outer epidermis is accompanied by epidermal cracks, where bacteria can easily attach and gain access to root tissues (Supplementary Fig. S4 at *JXB* online; Dong *et al.*, 2003; Tyler and Triplett, 2008). Thus, similarly to leaves, promoter activity can be found in cells vulnerable to bacterial infection.

### Flg22 regulates lateral root growth and auxin distribution

Long-term treatment with flg22 leads to inhibition of root growth in wild-type seedlings (Gomez-Gomez *et al.*, 1999). This study was extended and it was observed that the flg22-dependent inhibition of root growth (Supplementary Fig. S5C at *JXB* online) was accompanied by a reduced number of LRs (Fig. 3E, F). As LR initiation is strongly dependent on auxin accumulation in the cells primed for LR outgrowth (Dubrovsky *et al.*, 2008), experiments were carried out to determine whether flg22 treatment might interfere with auxin distribution and maxima during root and LR growth. *DR5*::GFP (auxin-responsive GFP) lines were treated with flg22 and it was found that the auxin maxima in the LR primordia are reduced after 72h of flg22 treatment compared with the control line, which was mock treated during this period (Fig. 3G). In addition, in the flg22-treated *DR5*::GFP seedlings, GFP signals were observed in the root epidermal cells, which were not present in control lines (Fig. 3G). Thus, these data showed that flg22 influences auxin distribution in a cell type-specific manner. The ectopic up-regulation of auxin in the epidermal cells as well as the down-regulation of auxin in the LR primordia might contribute to the flg22-dependent inhibition of root and LR growth. This correlates with the identification of AT1G59500 and AT1G68765 from the transcriptome data set, which are known auxin-responsive genes [The Arabidopsis Information Resource (TAIR)], and is in agreement with previous studies showing that auxin and auxin-responsive genes are also regulated by flg22 (Zipfel *et al.*, 2004; Navarro *et al.*, 2006). The findings are also consistent with reduced DR5–GUS expression in roots and inhibition of auxin-mediated adventitious root growth when triggered with oligogalacturonides, components of the plant cell wall known to trigger plant defences similar to PAMPs (Savatin *et al.*, 2011).

### Hormones and stress signals regulate *FLS2* expression in different root tissues

**Fig. 4.**
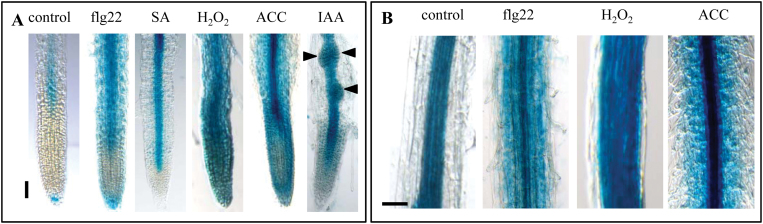
Induced *FLS2* expression in roots is regulated in a tissue-dependent manner. (A) Promoter activity in the root tip of *pFLS2*::GUS seedlings (8 d after germination) after treatment with flg22 (10 μM), SA (50 μM), H_2_O_2_ (1mM), ACC (10 μM), and IAA (10 μM). (B) Promoter activity in the root differentiation zone after flg22 (10 μM), H_2_O_2_ (1mM), and ACC (10 μM) treatment; (A, B) bar=100 μm.

**Fig. 5.**
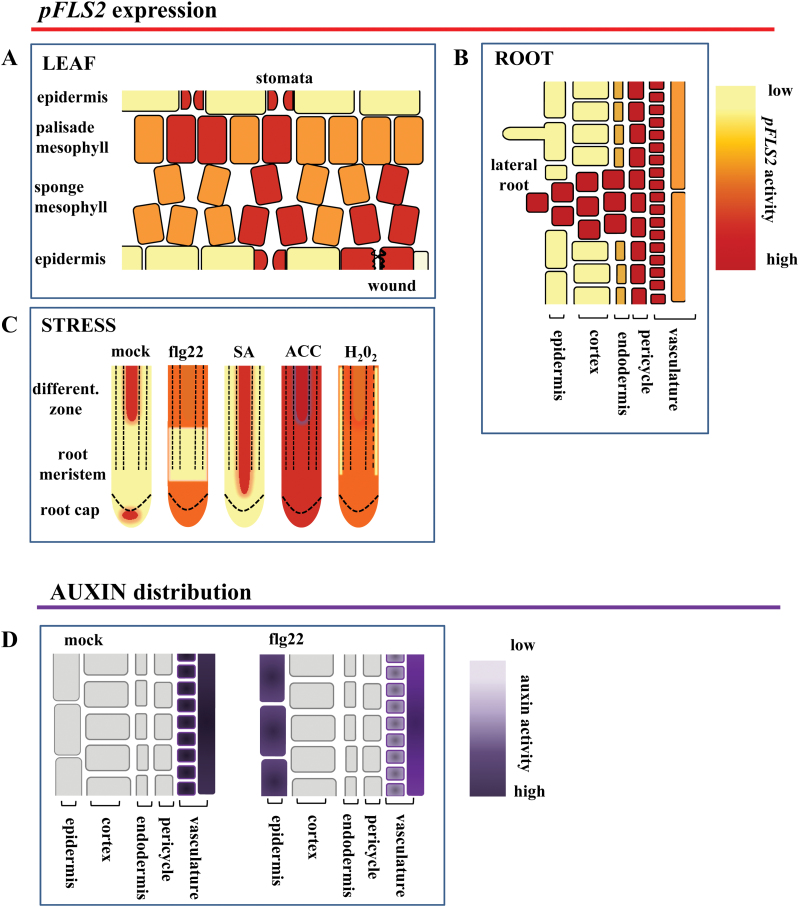
Model summarizing FLS2 cell-type and tissue-specific expression patterns. The cartoon depicts the promoter activity of *FLS2* in leaves (A) and roots (B); (C) stress responsiveness of the promoter in roots; and (D) flg22-dependent ectopic up-regulation of auxin in root epidermal cells.

PAMP-triggered immunity (PTI) is highly regulated by the action of phytohormones such as salicylic acid (SA), ethylene, and jasmonate (JA) (Bari and Jones, 2009). In this context, the different hormones and abiotic stresses which are known to play important roles in PTI responses were studied for their effect on *FLS2* promoter activity. In mock-treated roots, *pFLS2*::GUS expression was visible in the root late elongation zone, as described above (Figs 2A, 5). Additionally in ~20% of the control roots, a distinct GUS staining in root cap cells directly underneath the root meristem was observed (Fig. 4A). Incubation with flg22 led to an increased *FLS2* promoter activity in the root tip starting at the transition zone and extending to cortical cells in the differentiation zone (Fig. 4A, B). When treated with SA, *pFLS2::GUS* roots showed a strong blue staining in the vasculature, which started close to the meristematic zone (Fig. 4A), but did not extend to the cortex or to the differentiation zone (data not shown). Treatment with H_2_O_2_ or the ethylene precursor ACC provoked an almost uniform promoter activity in the root cap, root meristem, and root epidermal cells (Fig. 4A, B). However, ACC induced *pFLS2*:*:GUS* activity in the vasculature to a much higher extent compared with H_2_O_2_ or mock treatment (Fig. 4B).

It was also tested whether the promoter of *FLS2* is auxin responsive. The emergence of LR primordia becomes highly induced by incubation with the auxin analogue IAA, which exhibited clear *FLS2* promoter activity (Fig. 4A). However, GUS accumulation was specific to LR primordia and the vasculature in IAA-treated roots, and no GUS staining was observed in cortical cells. These experiments revealed that flg22, SA, H_2_O_2_, and ethylene all influence the expression activity of the *FLS2* promoter, but the responses are specific to different tissue layers in the root (Fig. 4). In summary, this identifies an unexpected level of tissue-dependent regulation of *FLS2* expression in response to a variety of different stresses (Fig. 5).

## Discussion

The prevailing view in plant immunity is that all plant cells are capable of pathogen perception and initial defence responses. This would require constitutive expression of at least the primary sensors of the immune system. Based on plant-scale expression analysis, *FLS2* was found in all plant organs including flowers, leaves, stems, and roots (Gomez-Gomez and Boller, 2000; this study). However, cell type-specific responses might play an important role in the context of how plants initiate defence responses against potentially invasive pathogens, but do not fend off beneficial microbes that are often needed for plant growth especially in low nutrient conditions (Bulgarelli *et al.*, 2013). It was therefore proposed that the cellular and tissue location of immune components is essential to mount the appropriate defence responses, and that they should be best located at putative entry sites of pathogens to inhibit their invasion efficiently (Faulkner and Robatzek, 2012). In this study, the *FLS2* promoter activity was followed and it was found that while *FLS2* is generally expressed in all tissues, there are remarkable differences in the level of the expression regulated in a cell type-specific and developmental manner. In addition, the *FLS2* promoter activity is responsive to several hormones playing roles in plant immunity such as SA and ethylene, which themselves are induced upon flg22 elicitation (Felix *et al.*, 1999; Tsuda *et al.*, 2008). Consistently, these and the present observations show that FLS2 is subject to positive regulation between receptor expression and the immune response.

### Prominent entry sites of potential pathogens are guarded by high *FLS2* expression

Hydathodes are pores at the leaf margin that are continuous with the xylem. Hydathodes are targeted by pathogenic bacteria such as *Xanthomonas campestris* pv. *campestris*, as points of access into plant tissue (Hugouvieux *et al.*, 1998). The stomatal pores (Zeng and He, 2010) represent another prominent entry route of bacterial pathogens. Stomata close upon PAMP perception to restrict pathogen entry, and successful pathogens secrete effectors such as HopM1, syringoline, and coronatine that inhibit the closure and/or actively induce re-opening (Melotto *et al.*, 2006; Schellenberg *et al.*, 2010; Zeng and He, 2010; Lozano-Duran *et al.*, 2014). Both cell types are characterized by a high promoter activity of *FLS2* compared with the surrounding mesophyll cells (Fig. 1C, E), suggesting that cells at tissue entry points are particularly well equipped to detect invading pathogens. Further, the mesophyll cells forming the substomatal cavity also exhibit a higher *FLS2* promoter activity (Figs 1F, 5). Previous data show that FLS2 mediates immunity at the level of stomatal entry (Zipfel *et al.*, 2004; Zeng and He, 2010). In agreement with this, stomatal expression of *FLS2* is enhanced upon bacterial infection. GUS staining is more intense in guard cells relative to the surrounding cells, indicating a guard cell-specific regulation of *FLS2* promoter activity (Fig. 1H). Although the possibility that the prominent GUS staining at hydathodes might be unspecific cannot be ruled out, *FLS2* expression at this location is consistent with the fact that hydathodes mark the end-points of the vasculature, another tissue exhibiting high *FLS2* expression and, importantly, responsive to flg22. The overall patterns of *pFLS2*::GUS expression observed (Fig. 5A–C) are in agreement with publicly available expression data (eFPBrowser; Faulkner and Robatzek, 2012).

Wounds and cracks in the epidermal layers represent sites of vulnerability with respect to pathogen infection. The bacterial colonization beyond these primary infection sites is dependent on secreted effectors such as syringoline promoting distant tissue colonization (Misas-Villamil *et al.*, 2013). The *FLS2* promoter is responsive to wounding in leaves (Fig. 1I; Supplementary Fig. S3C at *JXB* online), suggesting that cells at these sites might depend on higher FLS2 levels to fend off pathogen invasion of neighbouring tissues. This is consistent with a previous study which revealed that higher protein levels of FLS2 contributes to more flg22 binding and are positively associated with reduced *Pto* DC3000 proliferation (Vetter *et al.*, 2012).

Plants also have ‘natural’ wounds, which occur during the emergence of LRs. These manifest as ruptures in the epidermal cell layer around the LR meristem. Detailed observations of bacterial colonization of roots led to the assumption that bacteria use these LR emergence sites as entry routes to the roots (Dong *et al.*, 2003; Tyler and Triplett, 2008) (Supplementary Fig. S4B at *JXB* online). Although in developed roots *FLS2* expression was not present in the meristem, the *FLS2* promoter exhibited a strong activity in the LR primordia and outgrowing LRs (Fig. 3A–D). These observations indicate that the FLS2 expression is highly dynamic and regulated in a cell type- and development-dependent manner (Fig. 5B, C). Considering that LRs do not possess a root cap, which can also function as a PAMP-reactive physical barrier to the root meristem (Plancot *et al.*, 2013), it might be essential for a plant to guard the LR meristem.

### The vasculature is a tissue with high *FLS2* expression

Evidently, the vasculature provides excellent means for pathogens to spread throughout the plant. Together with the vasculature being rich in nutrients and water, this makes the vasculature a very attractive target tissue for pathogens. In plant interactions with a fungal pathogen, strong lignification of vascular bundles is associated with a compromised infection (Tanaka *et al.*, 2014). One significant observation of the present study is the defined and high activity of the *FLS2* promoter in the root stele, which is correlated with a high abundance of the FLS2–GFP fusion protein (Fig. 2B–E). Interestingly, high promoter activity in vascular tissue was also found for *PEPR1* and *PEPR2*, receptors associated with damage-elicited responses and immunity (Bartels *et al.*, 2013). In addition, it was observed that the vasculature contributes to the flg22-induced increase in [Ca^2+^]_i_ (Fig. 2F). It has been described that flg22 induces the production of lignin (Schenke *et al.*, 2011), but whether lignification is part of the FLS2-mediated immunity to prevent colonization and spread through the vasculature remains to be addressed. In the leaf, *Pseudomonas* bacteria colonize distant tissues along the vasculature (Misas-Villamil *et al.*, 2011), whereas, in the root, the bacterial pathogen *Ralstonia solanacearum* directly utilizes plant xylem vessels to move through the plant (Digonnet *et al.*, 2012). It is tempting to speculate that the absence of elicitor-active flagellin promotes the infection success of *R. solanacearum* bypassing FLS2-mediated defences in the vasculature (Pfund *et al.*, 2004).

While *FLS2* expression is restricted to the stele under normal conditions, expression can be expanded to the cortex under certain stresses (Figs 4, 5C) and it is shown that roots are sensitive to flg22 initiating typical defence responses (Millet *et al.*, 2010; Jacobs *et al.*, 2011; this study). It is possible that low expression of FLS2 in the root cortex allows the colonization of this tissue by beneficial bacteria without triggering defence. High constitutive expression of FLS2 in the stele might provide an additional barrier to bacterial invasion of the vascular tissue beyond the cortex, and stress-induced expansion of this zone of expression might reflect increased vulnerability of the tissue. Flg22-dependent gene induction was quite specifically activated in the elongation zone, whereas flg22-induced callose deposition was observed over the entire root length (Millet *et al.*, 2010). However, whether these immune response are initiated in epidermal cells, cortical cells, or inner cylinder cells needs to be addressed in the future.

### Auxin-mediated root development is responsive to flg22

The long-term incubation with flg22 is known to inhibit root growth (Gomez-Gomez *et al.*, 1999) and this inhibition of root growth is accompanied by a reduced development of LRs (Fig. 3E, F). Auxin, an important plant hormone involved in the regulation of root cell elongation and LR outgrowth, is found to be ectopically up-regulated in the epidermal cells of flg22-treated roots, whereas it is down-regulated in the LR primordia (Figs 3G, 5D). This is in agreement with studies describing an flg22-dependent antagonism for auxin activity, which leads to a rapid down-regulation of auxin-responsive genes and contributes to plant resistance against bacteria (Navarro *et al.*, 2006). Ectopic up-regulation of auxin in root epidermal cells was also described to be involved in ethylene-dependent root growth arrest (Ruzicka *et al.*, 2007). As ethylene production is triggered by flg22 (Felix *et al.*, 1999), it might be possible that these hormones are together integrated in the flg22-induced inhibition of root growth, with a possible outcome being that flg22 reduces putative bacterial entry points at LRs.

This interplay between the flg22 responses and hormone signalling is also reflected at the level of the *FLS2* promoter activity, as seen by the influence of IAA and ACC on the expression of *FLS2*. ACC treatment as well as the high induction around wound sites is consistent with a direct control of *FLS2* transcription by ethylene signalling (Boutrot *et al.*, 2010; Mersmann *et al.*, 2010). Altogether, these findings show a positive regulation of *FLS2* expression by hormones (ethylene and SA) and small signalling molecules such as reactive oxygen species, which are produced upon flg22 trigger (Bari and Jones, 2009). This positive transcriptional regulation might be important to deliver newly synthesized receptors to the plasma membrane since activated FLS2 is removed from the plasma membrane by endocytosis and degradation (Robatzek *et al.*, 2006; Göhre *et al.*, 2008; Smith *et al.*, 2014).

### Concluding remark

It is shown that the *FLS2* promoter activity maps to vulnerable tissue targeted by bacteria for entry and colonization in plants. This will be useful to understand the tissue- and cell type-specific role of FLS2 in immune signalling, and will aid in strategies to enhance plant resistance by targeting of defence to relevant tissues.

## Supplementary data

Supplementary data are available at *JXB* online.

Table S1. Flg22-up-regulated genes in roots; microarray data.

Figure S1. Prediction of *FLS2* promoter motifs 1000bp upstream of At5g46330.

Figure S2. *FLS2* promoter activity during plant development.

Figure S3. *FLS2* promoter activity during leaf development, wound stress. and biotic stress.

Figure S4. *Pto* DC3000–GFP localization on leaves and roots.

Figure S5. *FLS2* promoter activity in non-sterile grown roots and flg22-dependent inhibition of root growth.

Supplementary Data

## References

[CIT0001] Alassimone J Naseer S Geldner N 2010 A developmental framework for endodermal differentiation and polarity. Proceedings of the National Academy of Sciences, USA 107, 5214–5219. 10.1073/pnas.0910772107 PMC2841941 20142472

[CIT0002] Bari R Jones JD 2009 Role of plant hormones in plant defence responses. Plant Molecular Biology 69, 473–488. 19083153 10.1007/s11103-008-9435-0

[CIT0003] Bartels S Lori M Mbengue M van Verk M Klauser D Hander T Boni R Robatzek S Boller T 2013 The family of Peps and their precursors in Arabidopsis: differential expression and localization but similar induction of pattern-triggered immune responses. Journal of Experimental Botany 64, 5309–5321. 24151300 10.1093/jxb/ert330

[CIT0004] Benkova E Michniewicz M Sauer M Teichmann T Seifertova D Jurgens G Friml J 2003 Local, efflux-dependent auxin gradients as a common module for plant organ formation. Cell 115, 591–602. 14651850 10.1016/s0092-8674(03)00924-3

[CIT0005] Blume B Nurnberger T Nass N Scheel D 2000 Receptor-mediated increase in cytoplasmic free calcium required for activation of pathogen defense in parsley. The Plant Cell 12, 1425–1440. 10948260 10.1105/tpc.12.8.1425 PMC149113

[CIT0006] Boller T Felix G 2009 A renaissance of elicitors: perception of microbe-associated molecular patterns and danger signals by pattern-recognition receptors. Annual Review of Plant Biology 60, 379–406. 10.1146/annurev.arplant.57.032905.105346 19400727

[CIT0007] Boutrot F Segonzac C Chang KN Qiao H Ecker JR Zipfel C Rathjen JP 2010 Direct transcriptional control of the Arabidopsis immune receptor FLS2 by the ethylene-dependent transcription factors EIN3 and EIL1. Proceedings of the National Academy of Sciences, USA 107, 14502–14507. 10.1073/pnas.1003347107 PMC2922558 20663954

[CIT0008] Bulgarelli D Schlaeppi K Spaepen S Ver Loren van Themaat E Schulze-Lefert P 2013 Structure and functions of the bacterial microbiota of plants. Annual Review of Plant Biology 64, 807–838. 10.1146/annurev-arplant-050312-120106 23373698

[CIT0009] Chinchilla D Bauer Z Regenass M Boller T Felix G 2006 The Arabidopsis receptor kinase FLS2 binds flg22 and determines the specificity of flagellin perception. The Plant Cell 18, 465–476. 16377758 10.1105/tpc.105.036574 PMC1356552

[CIT0010] Clough SJ Bent AF 1998 Floral dip: a simplified method for Agrobacterium-mediated transformation of Arabidopsis thaliana. The Plant Journal 16, 735–743. 10069079 10.1046/j.1365-313x.1998.00343.x

[CIT0011] De Block M Debrouwer D 1992 *In-situ* enzyme histochemistry on plastic-embedded plant material. The development of an artefact-free P-glucuronidase assay. The Plant Journal 2, 261–266

[CIT0012] Digonnet C Martinez Y Denance N Chasseray M Dabos P Ranocha P Marco Y Jauneau A Goffner D 2012 Deciphering the route of Ralstonia solanacearum colonization in Arabidopsis thaliana roots during a compatible interaction: focus at the plant cell wall. Planta 236, 1419–1431. 22729825 10.1007/s00425-012-1694-y

[CIT0013] Dong Y Iniguez AL Ahmer BM Triplett EW 2003 Kinetics and strain specificity of rhizosphere and endophytic colonization by enteric bacteria on seedlings of Medicago sativa and Medicago truncatula. Applied Environmental Microbiology 69, 1783–1790. 12620870 10.1128/AEM.69.3.1783-1790.2003 PMC150109

[CIT0014] Dubrovsky JG Sauer M Napsucialy-Mendivil S Ivanchenko MG Friml J Shishkova S Celenza J Benkova E 2008 Auxin acts as a local morphogenetic trigger to specify lateral root founder cells. Proceedings of the National Academy of Sciences, USA 105, 8790–8794. 10.1073/pnas.0712307105 PMC2438385 18559858

[CIT0015] Faulkner C Robatzek S 2012 Plants and pathogens: putting infection strategies and defence mechanisms on the map. Current Opinion in Plant Biology 15, 699–707. 22981427 10.1016/j.pbi.2012.08.009

[CIT0016] Felix G Duran JD Volko S Boller T 1999 Plants have a sensitive perception system for the most conserved domain of bacterial flagellin. The Plant Journal 18, 265–276. 10377992 10.1046/j.1365-313x.1999.00265.x

[CIT0017] Fricker MD Plieth C Knight H Blancaflor E Knight MR White NS Gilroy S 1999 Fluorescence and luminescence techniques to probe ion activities in living plant cells. In: Mason WT, ed. Fluorescent and luminescent probes for biological activity, 2nd edn. Academic Press, 569–596.

[CIT0018] Göhre V Spallek T Häweker H Mersmann S Mentzel T Boller T de Torres M Mansfield JW Robatzek S 2008 Plant pattern-recognition receptor FLS2 is directed for degradation by the bacterial ubiquitin ligase AvrPtoB. Current Biology 18, 1824–1832. 19062288 10.1016/j.cub.2008.10.063

[CIT0019] Gomez-Gomez L Boller T 2000 FLS2: an LRR receptor-like kinase involved in the perception of the bacterial elicitor flagellin in Arabidopsis. Molecular Cell 5, 1003–1011. 10911994 10.1016/s1097-2765(00)80265-8

[CIT0020] Gomez-Gomez L Felix G Boller T 1999 A single locus determines sensitivity to bacterial flagellin in Arabidopsis thaliana. The Plant Journal 18, 277–284. 10377993 10.1046/j.1365-313x.1999.00451.x

[CIT0021] Häweker H Rips S Koiwa H Salomon S Saijo Y Chinchilla D Robatzek S von Schaewen A 2010 Pattern recognition receptors require N-glycosylation to mediate plant immunity. Journal of Biological Chemistry 12, 4629–4636. 20007973 10.1074/jbc.M109.063073 PMC2836068

[CIT0022] Hugouvieux V Barber CE Daniels MJ 1998 Entry of Xanthomonas campestris pv. campestris into hydathodes of Arabidopsis thaliana leaves: a system for studying early infection events in bacterial pathogenesis. Molecular Plant-Microbe Interactions 11, 537–543. 9612952 10.1094/MPMI.1998.11.6.537

[CIT0023] Jacobs S Zechmann B Molitor A Trujillo M Petutschnig E Lipka V Kogel KH Schafer P 2011 Broad-spectrum suppression of innate immunity is required for colonization of Arabidopsis roots by the fungus Piriformospora indica. Plant Physiology 156, 726–740. 21474434 10.1104/pp.111.176446 PMC3177271

[CIT0024] Lozano-Duran R Bourdais G He SY Robatzek S 2014 The bacterial effector HopM1 suppresses PAMP-triggered oxidative burst and stomatal immunity. New Phytologist 202, 259–269. 24372399 10.1111/nph.12651

[CIT0025] Lozano-Duran R Macho AP Boutrot F Segonzac C Somssich IE Zipfel C 2013 The transcriptional regulator BZR1 mediates trade-off between plant innate immunity and growth. Elife 2, e00983. 24381244 10.7554/eLife.00983 PMC3875382

[CIT0026] Marti MC Stancombe MA Webb AA 2013 Cell- and stimulus type-specific intracellular free Ca2+ signals in Arabidopsis. Plant Physiology 163, 625–634. 24027243 10.1104/pp.113.222901 PMC3793043

[CIT0027] Melotto M Underwood W Koczan J Nomura K He SY 2006 Plant stomata function in innate immunity against bacterial invasion. Cell 126, 969–980. 16959575 10.1016/j.cell.2006.06.054

[CIT0028] Mersmann S Bourdais G Rietz S Robatzek S 2010 Ethylene signaling regulates accumulation of the FLS2 receptor and is required for the oxidative burst contributing to plant immunity. Plant Physiology 154, 391–400. 20592040 10.1104/pp.110.154567 PMC2938167

[CIT0029] Millet YA Danna CH Clay NK Songnuan W Simon MD Werck-Reichhart D Ausubel FM 2010 Innate immune responses activated in Arabidopsis roots by microbe-associated molecular patterns. The Plant Cell 22, 973–990. 20348432 10.1105/tpc.109.069658 PMC2861455

[CIT0030] Misas-Villamil JC Kolodziejek I Crabill E Kaschani F Niessen S Shindo T Kaiser M Alfano JR van der Hoorn RA 2013 Pseudomonas syringae pv. syringae uses proteasome inhibitor syringolin A to colonize from wound infection sites. PLoS Pathogenesis 9, e1003281. 10.1371/journal.ppat.1003281 PMC3610659 23555272

[CIT0031] Misas-Villamil JC Kolodziejek I van der Hoorn RA 2011 Pseudomonas syringae colonizes distant tissues in Nicotiana benthamiana through xylem vessels. The Plant Journal 67, 774–782. 21554458 10.1111/j.1365-313X.2011.04632.x

[CIT0032] Navarro L Dunoyer P Jay F Arnold B Dharmasiri N Estelle M Voinnet O Jones JD 2006 A plant miRNA contributes to antibacterial resistance by repressing auxin signaling. Science 312, 436–439. 16627744 10.1126/science.1126088

[CIT0033] Navarro L Jay F Nomura K He SY Voinnet O 2008 Suppression of the microRNA pathway by bacterial effector proteins. Science 321, 964–967. 18703740 10.1126/science.1159505 PMC2570098

[CIT0034] Pfund C Tans-Kersten J Dunning FM Alonso JM Ecker JR Allen C Bent AF 2004 Flagellin is not a major defense elicitor in Ralstonia solanacearum cells or extracts applied to Arabidopsis thaliana. Molecular Plant-Microbe Interactions 17, 696–706. 15195952 10.1094/MPMI.2004.17.6.696

[CIT0035] Plancot B Santaella C Jaber R Kiefer-Meyer MC Follet-Gueye ML Leprince J Gattin I Souc C Driouich A Vicre-Gibouin M 2013 Deciphering the responses of root border-like cells of Arabidopsis and flax to pathogen-derived elicitors. Plant Physiology 163, 1584–1597. 24130195 10.1104/pp.113.222356 PMC3850203

[CIT0036] Preston GM 2000 Pseudomonas syringae pv. tomato: the right pathogen, of the right plant, at the right time. Molecular Plant Pathology 1, 263–275. 20572973 10.1046/j.1364-3703.2000.00036.x

[CIT0037] Ranf S Wunnenberg P Lee J Becker D Dunkel M Hedrich R Scheel D Dietrich P 2008 Loss of the vacuolar cation channel, AtTPC1, does not impair Ca2+ signals induced by abiotic and biotic stresses. The Plant Journal 53, 287–299. 18028262 10.1111/j.1365-313X.2007.03342.x

[CIT0038] Robatzek S Chinchilla D Boller T 2006 Ligand-induced endocytosis of the pattern recognition receptor FLS2 in Arabidopsis. Genes and Development 20, 537–542. 16510871 10.1101/gad.366506 PMC1410809

[CIT0039] Ruzicka K Ljung K Vanneste S Podhorska R Beeckman T Friml J Benkova E 2007 Ethylene regulates root growth through effects on auxin biosynthesis and transport-dependent auxin distribution. The Plant Cell 19, 2197–2212. 17630274 10.1105/tpc.107.052126 PMC1955700

[CIT0040] Savatin DV Ferrari S Sicilia F De Lorenzo G 2011 Oligogalacturonide–auxin antagonism does not require posttranscriptional gene silencing or stabilization of auxin response repressors in Arabidopsis. Plant Physiology 157, 1163–1174. 21880931 10.1104/pp.111.184663 PMC3252154

[CIT0041] Schellenberg B Ramel C Dudler R 2010 Pseudomonas syringae virulence factor syringolin A counteracts stomatal immunity by proteasome inhibition. Molecular Plant-Microbe Interactions 23, 1287–1293. 20831408 10.1094/MPMI-04-10-0094

[CIT0042] Schenke D Bottcher C Scheel D 2011 Crosstalk between abiotic ultraviolet-B stress and biotic (flg22) stress signalling in Arabidopsis prevents flavonol accumulation in favor of pathogen defence compound production. Plant, Cell and Environment 34, 1849–1864. 10.1111/j.1365-3040.2011.02381.x 21707654

[CIT0043] Smith JM Salamango DJ Leslie ME Collins CA Heese A 2014 Sensitivity to Flg22 is modulated by ligand-induced degradation and de novo synthesis of the endogenous flagellin-receptor FLAGELLIN-SENSING2. Plant Physiology 164, 440–454. 24220680 10.1104/pp.113.229179 PMC3875820

[CIT0044] Tanaka S Brefort T Neidig N Djamei A Kahnt J Vermerris W Koenig S Feussner K Feussner I Kahmann R 2014 A secreted Ustilago maydis effector promotes virulence by targeting anthocyanin biosynthesis in maize. Elife 3, e01355. 24473076 10.7554/eLife.01355 PMC3904489

[CIT0045] Tsuda K Sato M Glazebrook J Cohen JD Katagiri F 2008 Interplay between MAMP-triggered and SA-mediated defense responses. The Plant Journal 53, 763–775. 18005228 10.1111/j.1365-313X.2007.03369.x

[CIT0046] Tyler HL Triplett EW 2008 Plants as a habitat for beneficial and/or human pathogenic bacteria. Annual Review of Phytopathology 46, 53–73. 10.1146/annurev.phyto.011708.103102 18680423

[CIT0047] Vetter MM Kronholm I He F Haweker H Reymond M Bergelson J Robatzek S de Meaux J 2012 Flagellin perception varies quantitatively in Arabidopsis thaliana and its relatives. Molecular Biology and Evolution 29, 1655–1667. 22319159 10.1093/molbev/mss011

[CIT0048] Zeng W He SY 2010 A prominent role of the flagellin receptor FLAGELLIN-SENSING2 in mediating stomatal response to Pseudomonas syringae pv tomato DC3000 in Arabidopsis. Plant Physiology 153, 1188–1198. 20457804 10.1104/pp.110.157016 PMC2899927

[CIT0049] Zipfel C Kunze G Chinchilla D Caniard A Jones JDG Boller T Felix G 2006 Perception of the bacterial PAMP EF-Tu by the receptor EFR restricts Agrobacterium-mediated transformation. Cell 125, 749–760. 16713565 10.1016/j.cell.2006.03.037

[CIT0050] Zipfel C Robatzek S Navarro L Oakeley EJ Jones JD Felix G Boller T 2004 Bacterial disease resistance in Arabidopsis through flagellin perception. Nature 428, 764–767. 15085136 10.1038/nature02485

