# Supplementary material for: Expression patterns of FLAGELLIN SENSING 2 map to bacterial entry sites in plant shoots and roots
Source: J Exp Bot. 2014 Sep 9;65(22):6487–98. doi: 10.1093/jxb/eru366 (PMC4246182; doi:10.1093/jxb/eru366)
Supplement: Supplementary Data [file supp_65_22_6487__index.html]

Expression patterns of FLAGELLIN SENSING 2 map to bacterial entry sites in plant shoots and roots — Expression patterns of FLAGELLIN SENSING 2 map to bacterial entry sites in plant shoots and roots — Supplementary Data 

# Expression patterns of *FLAGELLIN SENSING 2* map to bacterial entry sites in plant shoots and roots

## Supplementary Data

Data files

**Files in this Data Supplement:**

- Supplementary Data - Supplementary Data
- Supplementary Data - Supplementary Data
