# Supplementary material for: Expression patterns of FLAGELLIN SENSING 2 map to bacterial entry sites in plant shoots and roots
Source: J Exp Bot. 2014 Sep 9;65(22):6487–98. doi: 10.1093/jxb/eru366 (PMC4246182; doi:10.1093/jxb/eru366)
Supplement: Supplementary Data [file supp_eru366_jexbot125708_file001.pdf]

Supplementary data:

Expression patterns of *FLAGELLIN SENSING 2* map to bacterial entry sites in plant shoots and roots

Martina Beck[1], *Ines Wyrsch*[2], *James Strutt*[1], *Rinukshi Wimalasekera*[3], *Alex Webb*[3], *Thomas Boller*[2] *and Silke Robatzek*[1]

[1]The Sainsbury Laboratory, Norwich Research Park, Norwich, NR4 7UH, UK

[2]Zürich-Basel Plant Science Center; University of Basel; Department of Environmental Sciences; Botany; Basel, Switzerland

[3]Department of Plant Sciences, University of Cambridge, Downing Street, Cambridge CB2 3EA, UK

Supplement Table*:*

Table 1: Flg22 up-regulated genes in roots, microarray data

Supplement Figures*:*

Fig S1: Prediction of *FLS2* promoter motifs 1000bp upstream of At5g46330

(A) Visualization of promoter motifs 1000 bp upstream of ATG. **(B)** Details of predicted promoter motifs 1000 bp upstream of ATG.

Prediction with Athena: a resource for rapid visualization and systematic analysis of Arabidopsis promoter sequences (O'Connor *et al.,* 2005; http://www.bioinformatics2.wsu.edu/Athena).

Figure S2: *FLS2* promoter activity during plant development

pFLS2::*GUS* is ubiquitously expressed in **(A)** 2 days-old seedling, in **(B)** 8 days-old seedling cotyledons and **(C)** hypocotyl; and highly expressed in **(D)** flower sepals and **(E)** stamen, in **(F)** pod dehiscence zone in mature siliques and **(G)** stipules.

Figure S3: *FLS2* promoter activity during leaf development, wound stress and biotic stress

(A) Definition of different leaf stages; **(B)** GUS staining of *pFLS2*::*GUS* in different leaf stages. Insert shows hydathodes. **(C)** Wound-induced promoter activity in different leaf stages. Insert shows hydathodes. **(D)** Hypocotyl after *Pto* DC3000 incubation with enhanced promoter activity in stomata (arrows), compared to mock treated (10mM $MgCl_2$) hypocotyl; bar = 100 µm.

Figure S4: *Pto* DC3000-GFP localization on leaves and roots

(A) Confocal micrographs showing *Pto* DC3000-GFP (green) on Col-0 leaves, upper panel shows bacteria on epidermis, encircled are stomata, lower panel shows bacteria (arrow heads) in the apoplast of mesophyll cells (block arrows); chloroplast autofluorescence is represented in red. **(B)** Confocal micrographs showing DC3000-GFP (green) bacteria on Col-0 roots stained with propidium iodide (red); note the accumulation of bacteria (arrow heads) at outgrowing LR (block arrows).

Figure S5: *FLS2* promoter activity in non-sterile grown roots and flg22 dependent inhibition of root growth

(A) *pFLS2*::*GUS* activity in 14 days-old seedlings grown on soil, white arrow shows inner vasculature, black arrows mark expansion of GUS staining, bar = 50 µm. **(B)** Cross section of roots grown on soil, arrows mark expanded GUS activity in endodermis, bar = 10 µm. **(C)** Graph depicts root length of 12 days-old Col-0 or *fls2* treated with 1 µM flg22 or without (same seedlings as used for 3F, bars represent average of 3 independent experiments, error bars represent SD, statistical significance represented with student t-test (p-value >0.001).

A

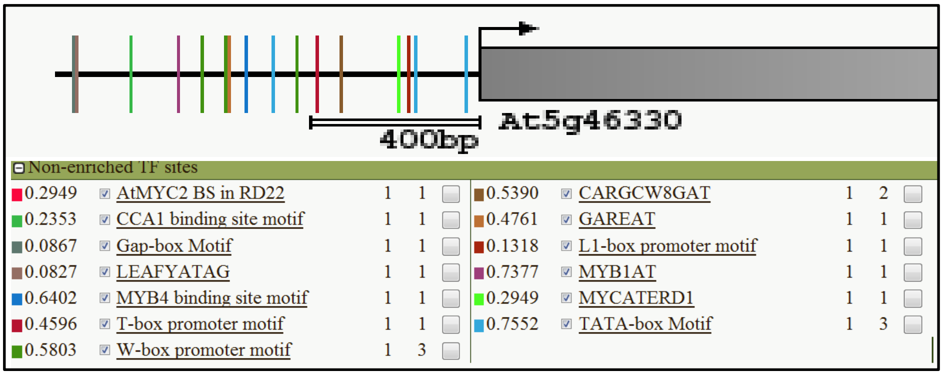

B

**At5g46330**		
Motifs		
**AtMYC2 BS in RD22**		**CACATG**
189 -184	(-)	CATGTG
**CARGCW8GAT**		**CWWWWWWWWG**
329 -320	(+)	CATAAATAAG
329 -320	(-)	CATAAATAAG
**CCA1 binding site motif**		**AAMAATCT**
826 -819	(-)	AGATTGTT
**GAREAT**		**TAACAAR**
593 -587	(+)	TAACAAA
**Gap-box Motif**		**CAAATGAA**
962 -955	(-)	TTCATTTG
**L1-box promoter motif**		**TAAATGYA**
164 -157	(-)	TACATTTA
**LEAFYATAG**		**CCAATGT**
953 -947	(+)	CCAATGT
**MYB1AT**		**WAACCA**
710 -705	(+)	AAACCA
**MYB4 binding site motif**		**AMCWAMC**
550 -544	(-)	GTTTGTT
**MYCATERD1**		**CATGTG**
189 -184	(+)	CATGTG
**T-box promoter motif**		**ACTTTG**
381 -376	(+)	ACTTTG
**TATA-box Motif**		**TATAAA**
484 -479	(+)	TATAAA
29 -24	(+)	TATAAA
151 -146	(-)	TTTATA
**W-box promoter motif**		**TTGACY**
601 -596	(+)	TTGACC
432 -427	(+)	TTGACT
655 -650	(-)	AGTCAA
**EIN3 binding sites**		**ATGTATCT**
325-317		ATGAATaT
304-297		AcATACAT
285-278		ATGAATaT

Figure S1

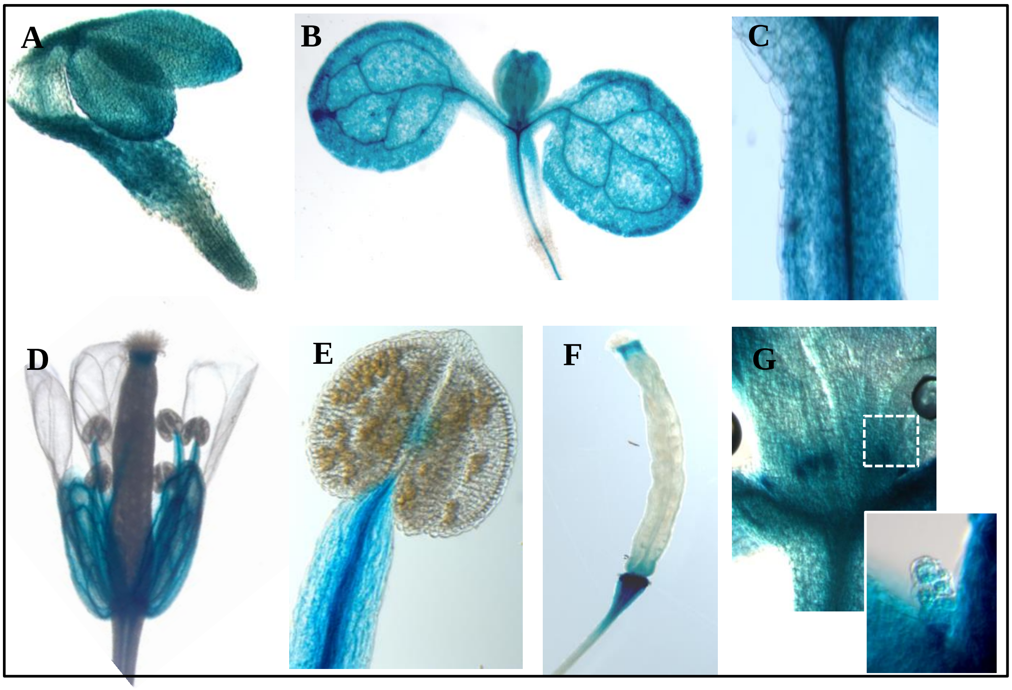

Figure S2

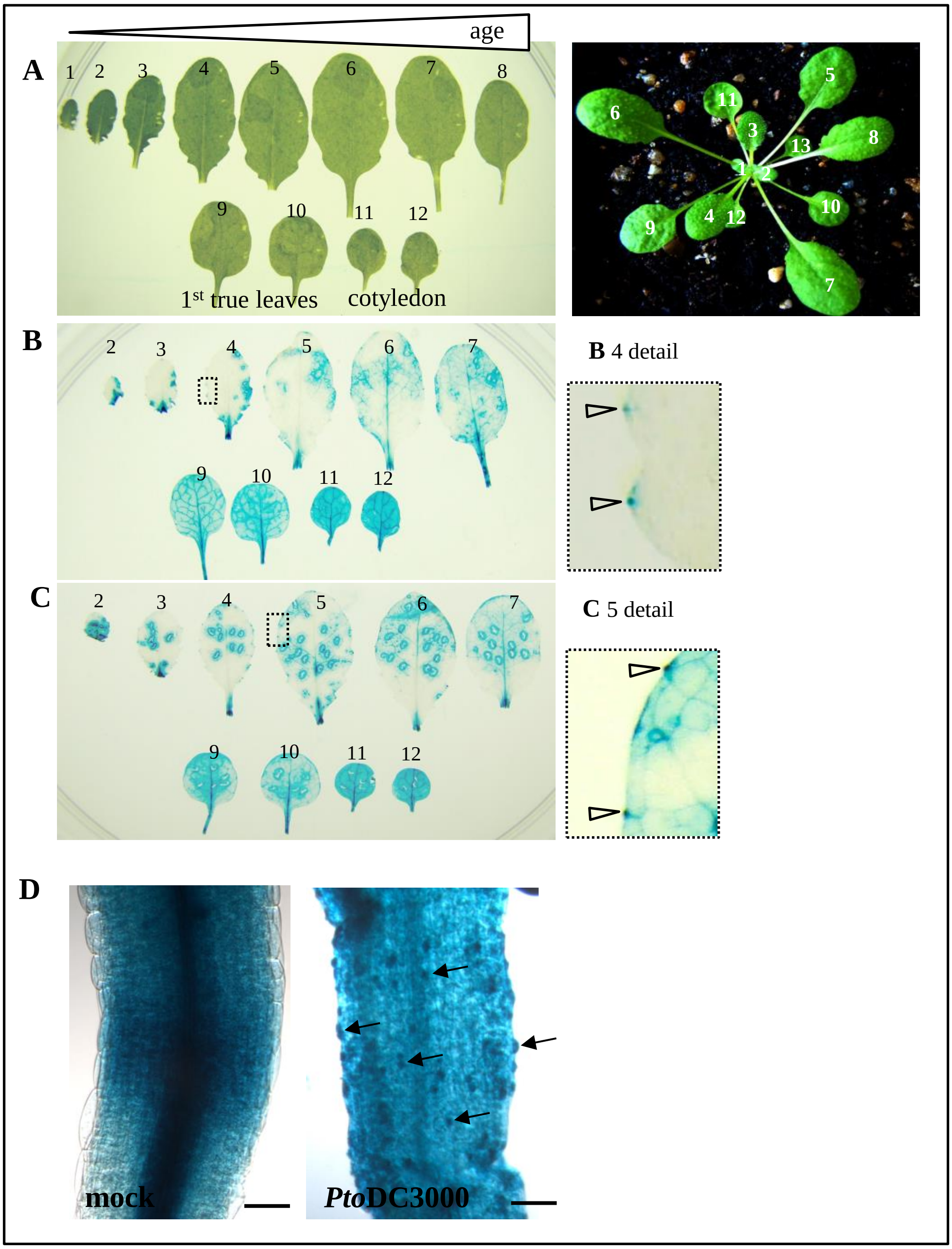
age
A
1
2
3
4
5
6
7
8
9
10
11
12
1st true leaves
cotyledon
6
11
5
3
13
8
1
2
4
12
10
9
7
B
B 4 detail
C
C 5 detail
D
mock
PtoDC3000

Figure S3

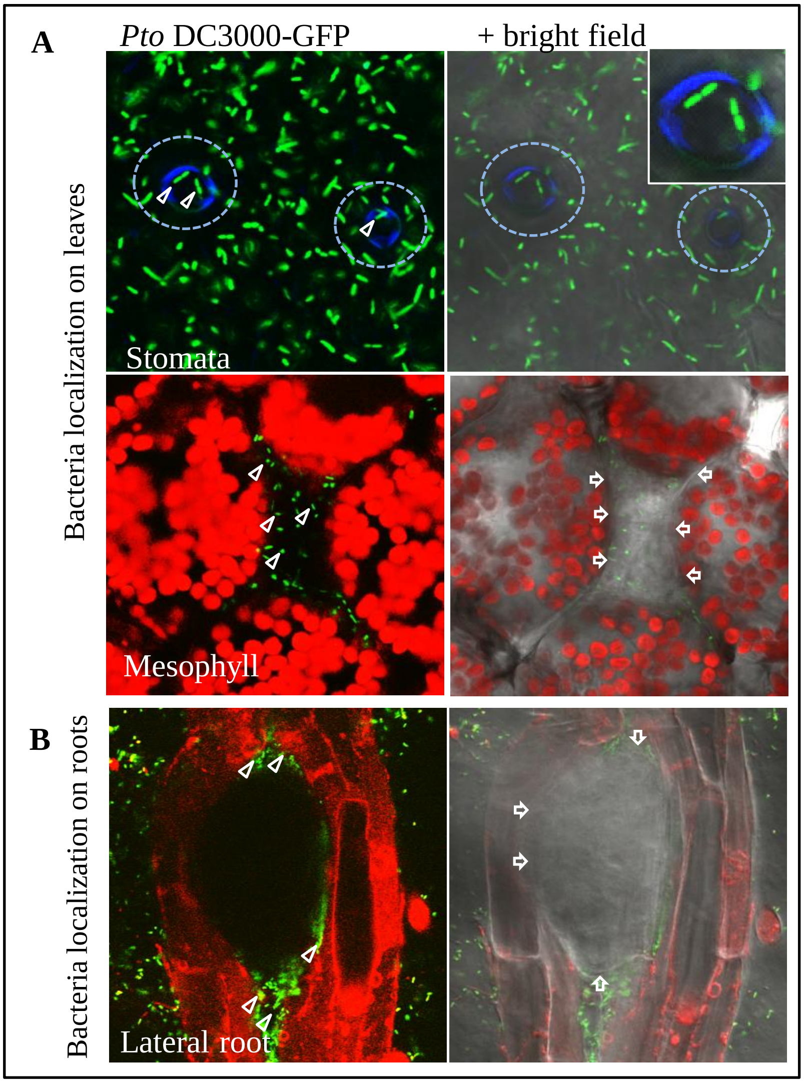
A
Pto DC3000-GFP
+ bright field
Bacteria localization on leaves
Stomata
Mesophyll
B
Bacteria localization on roots
Lateral root

Figure S4

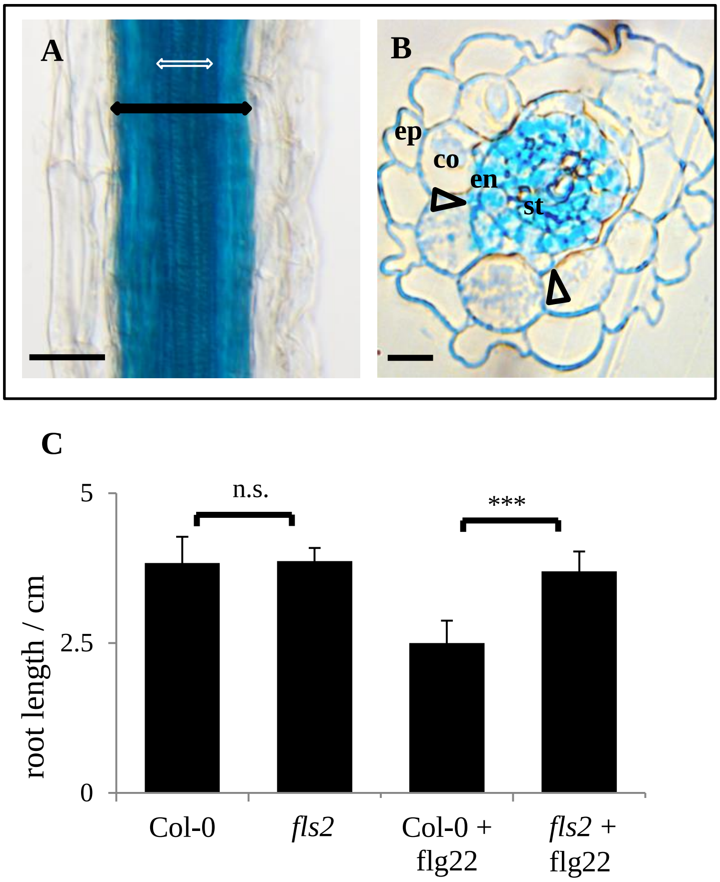
A
B
ep
co
en
st
C
root length / cm
5
2.5
0
n.s.
***
Col-0
fls2
Col-0 + flg22
fls2 + flg22

Figure S5
